# Reviewer Acknowledgement 2013

**DOI:** 10.1186/1471-2261-14-17

**Published:** 2014-02-13

**Authors:** Tim Shipley

**Affiliations:** 1 BioMed Central, Floor 6, 236 Gray's Inn Road, London WC1X 8HB, UK

## Abstract

**Contributing reviewers:**

The editors of BMC Cardiovascular Disorders would like to thank all our reviewers who have contributed to the journal in Volume 13 (2013).

## 

Jawdat Abdulla

Denmark

Suhad AbuMweis

Jordan

Akilew Adane

Ethiopia

Ismayil Ahmet

United States of America

Elena Aikawa

United States of America

Muharrem Akin

Germany

Thomas Alexander

India

Stephane Allouche

France

Wassim Almawi

Bahrain

Enrico Ammirati

Italy

Sina Andalib

Iran

Giuseppe Andò

Italy

Bruno Andrea

Brazil

Emiliano Angeloni

Italy

Uzair Ansari

Germany

Yaron Arbel

Israel

Carmelina Ariano

Italy

Ehrin Armstrong

United States of America

John Atherton

Australia

Vasilios Athyros

Greece

Ajax Atta

Brazil

Wael Attia

Egypt

Johann Auer

Austria

Ahmet Aykan

Turkey

Konstantinos Aznaouridis

Greece

Tetsuya Babazono

Japan

Edward Baker

New Zealand

Flavia Ballocca

Italy

Michael Balogun

Nigeria

Umberto Barbero

Italy

Geoffrey Barnes

United States of America

Marjorie Bastien

Canada

Ali Baykan

Turkey

Giorgio Bedogni

Italy

Miles Behan

United Kingdom

Iddo Ben-Dov

Israel

Robert Berent

Austria

Marius Berman

United Kingdom

Alejandro Berra

Argentina

Anna Bersano

Italy

Ami Bhatt

United States of America

Pawel Bieganowski

Poland

Georg Biesenbach

Austria

Grzegorz Bilo

Italy

Kåre I. Birkeland

Norway

Jacqueline Birks

United Kingdom

Utpal Kumar Biswas

India

Edward Bixler

United States of America

Federico Bizzarri

Italy

Florian Bönner

Germany

Emanuela Bostjancic

Slovenia

Marek Brabec

Czech Republic

Elizabeth Bradley

United States of America

Jerry Braun

Netherlands

Clinton Brawner

United States of America

Jana Brguljan Hitij

Slovenia

Jean-Christophe Brisset

United States of America

Andrew Burns

Australia

Michael Bursztyn

Israel

Rodrigo Calado

Brazil

Duncan Campbell

Australia

Gianluca Campo

Italy

Joseph Casey

Canada

Jasper Chan

Hong Kong

Xing-Zhen Chen

Canada

Shang-Jyh Chiou

Taiwan

Vineet Chopra

United States of America

Mirjam Christ-Crain

Switzerland

KR Julian Chun

Germany

Antonio Ciccarelli

Italy

Jose Alexandre Cipolli

Brazil

Neree Claes

Belgium

Bimmer Claessen

Netherlands

Paul Clift

United Kingdom

Hussain Contractor

United Kingdom

Rachael Cordina

United Kingdom

Michele Correale

Italy

Giovanni Corso

Italy

Nadege Costa

France

Lucia Costa-Paiva

Brazil

Xenophon Costeas

Greece

Thais Coutinho

United States of America

Magdalena Cuenca Garcia

Spain

Veronica D ' Annunzio

Argentina

Makoto Daimon

Japan

Francisco Darrieux

Brazil

Sarah Davis

United Kingdom

Arienne de Jong

Netherlands

Helma de Morree

Netherlands

Renato De Vecchis

Italy

Kurt Debl

Germany

Cristina Del-Ben

Brazil

Christian Delles

United Kingdom

Giuseppe Derosa

Italy

Yvan Devaux

Luxembourg

James DiNicolantonio

United States of America

Peter Dome

Hungary

Troy Dominguez

United Kingdom

Martin Donato

Argentina

Chiara Donfrancesco

Italy

Rachel Dreyer

United States of America

Yves d'Udekem

Australia

Joao Durigan

Brazil

Rita Durigan

Brazil

Orestis Efthimiou

Greece

Mats Eliasson

Sweden

Jeanette Erdmann

Germany

Paul Erne

Switzerland

Brigitte Escoubet

France

Rodrigo Estevez-Loureiro

Spain

Inmaculada Failde

Spain

Ayodele Falase

Nigeria

Oluranti Familoni

Nigeria

E. Vincent Faustino

United States of America

Nowell Fine

Canada

Sara Fitzgerald-Butt

United States of America

Bernard Fleury

France

Terrence Forrester

Jamaica

Gerry Fowkes

United Kingdom

Claude Franceschi

France

Nikolaos Frangogiannis

United States of America

Suzanne Fredericks

Canada

David Freedman

United States of America

Adelaide Fusco

Italy

Juan Gagliardi

Argentina

Seana Gall

Australia

Alexander Gallus

Australia

Luis García-Ortiz

Spain

Achille Gaspardone

Italy

Thomas Gaziano

United States of America

Francesca Giordana

Italy

Gunnar Gislason

Denmark

Betti Giusti

Italy

Luigi Gnudi

United Kingdom

Jens P. Goetze

Denmark

Phil Gona

United States of America

German Gonzalez

Argentina

Antonio Gotto

United States of America

Maria Grau

Spain

Maik J Grundeken

Netherlands

Maria Gueorguiev

United Kingdom

Mariann Gyongyosi

Austria

Majid Haghjoo

Iran

David Halon

Israel

Nobuyuki Hamajima

Japan

Naomi Hamburg

United States of America

Yasmin Hamirani

United States of America

Karen Harkness

Canada

Yonathan Hasin

Israel

Meian He

China

Jan Heeringa

Netherlands

Sean Heffron

United States of America

Andreas Henke

Germany

Eugene Hessel, II

United States of America

Willibald Hochholzer

Germany

Chung-Cheng Hsieh

United States of America

Ming-I Hsu

Taiwan

Pai Feng Hsu

Taiwan

Sandy Hu

China

Shi Hu

China

Gianluca Iacobellis

United States of America

Ignatios Ikonomidis

Greece

Fabio Infusino

Italy

Yasutaka Inuzuka

Japan

Hart Isaacs

United States of America

Kasper Iversen

Denmark

Yoshitaka Iwanaga

Japan

Sae Young Jae

Korea, South

Jiann-Shing Jeng

Taiwan

Alexander Jenson

United States of America

Myung Ho Jeong

Korea, South

Kandiah Jeyaseelan

Singapore

Zhi-Cheng Jing

China

Mayuko Kadono

Japan

Stephen Kaptoge

United Kingdom

Ganesan Karthikeyan

India

Tarek Kashour

Saudi Arabia

David Katerndahl

United States of America

Hiroaki Kawano

Japan

Aya Kawasaki

Japan

Nadia Khan

Canada

Amit Khera

United States of America

Madhu Khullar

India

Tuomas Kilpelainen

Denmark

Sang Wook Kim

Korea, South

Jolanda Kluin

Netherlands

Yoshio Kobayashi

Japan

Vasilios Kotsis

Greece

Martin Krssak

Austria

Iolanthe Kruger

South Africa

Mariusz Kruk

Poland

Kiyoshi Kume

Japan

Varant Kupelian

United States of America

Gaetano Lanza

Italy

Ioannis Laoutaris

Greece

John Lawrenson

South Africa

Won Young Lee

Korea, South

Gilles Lemesle

France

Albert Li

Hong Kong

Jian-Jun Li

China

Xiaotao Li

China

Clare Liddy

Canada

Hsuan-Hung Lin

Taiwan

Jan Lindeman

Netherlands

Songtao Liu

United States of America

Yanping Liu

Belgium

Jennifer Logue

United Kingdom

Carl Lombard

South Africa

Benjamin Longo-Mbenza

South Africa

Eric Loucks

United States of America

John Jenn-Yenn Lu

Taiwan

Carla Lubrano

Italy

George Lui

United States of America

Jared Magnani

United States of America

Manfred Maier

Austria

Olebogeng Majane

South Africa

Alberto Malerba

United Kingdom

Massimo Mancone

Italy

Nassos Manginas

Greece

Ferdinando Mannello

Italy

Warren Manning

United States of America

Antonis Manolis

Greece

Themistoklis Maounis

Greece

Serge Masson

Italy

Ruth Masterson Creber

United States of America

Anders Mathiasen

Denmark

Patrick Mathieu

Canada

John McClure

United Kingdom

Richard McNally

United Kingdom

Nehal Mehta

United States of America

Tracy Merlin

Australia

Antonio Miceli

Italy

Ashley M. Miller

American Samoa

Virginia M. Miller

United States of America

Carlos Miranda

Brazil

Karol Miszalski-Jamka

Poland

Xiumei Mo

China

Susanne Moebus

Germany

Deddo Moertl

Austria

Augusto Montezano

United Kingdom

Christian Mueller

Switzerland

Thomas Mueller

Austria

Thomas Muenzel

Germany

Maria Lorenza Muiesan

Italy

Sergio Nabais

Portugal

Kristina Nadrah

Slovenia

Kamatham Akhilender Naidu

India

Maria Navas

United States of America

Isao Nishi

Japan

Marko Noc

Slovenia

Constance Noguchi

United States of America

Youssef Nosir

Saudi Arabia

Argyrios Ntalianis

Greece

Gaetano Nucifora

Italy

Altacilio Nunes

Brazil

Slobodan Obradovic

Serbia

Teruo Okabe

Japan

Takafumi Okura

Japan

Adelaide Olson

United States of America

Koh Ono

Japan

Martin Orban

Germany

Christian Ott

Germany

Vittorio Palmieri

Italy

Constantinos Pantos

Greece

Jong-Seon Park

Korea, South

Jeetesh Patel

United Kingdom

Antonio Pazin-Filho

Brazil

Marco Perez

United States of America

John Perry

United Kingdom

Manuel Pestana

Portugal

Sanne A. E. Peters

Netherlands

Stefano Pidello

Italy

Rudin Pistulli

Germany

Dusan Popadic

Serbia

Zoran Popovic

United States of America

Tatjana Potpara

Serbia

Guido Pucciarelli

Italy

Aniket Puri

New Zealand

Giorgio Quadri

Italy

Natasa Rancic

Serbia

Thomas Rasmussen

Denmark

Rainer Rauramaa

Finland

Julie Redfern

Australia

Manja Reimann

Germany

Veronika Reinhard

Estonia

Manuel Rodriguez

Argentina

José Manuel Rodríguez-Pérez

Mexico

Shawn Rose

United States of America

Elaine Rush

New Zealand

Fraser Russell

Australia

Rafael Sadaba

Spain

Jason Salemi

United States of America

Gil Salles

Brazil

Carmine Savoia

Italy

Mark Schafer

United States of America

Michaela Louise Schiøtz

Denmark

Andre Schmidt

Brazil

Markus Schneider

Germany

Mikael Schneider

Denmark

Shaun Scholes

United Kingdom

Jenna Scisco

United States of America

Yackoob Seedat

South Africa

Jelena Seferovic

Serbia

Rasmus Sejersten Ripa

Denmark

Sharmini Selvarajah

Malaysia

Mario Sénéchal

Canada

Ankur Sethi

United States of America

Liang Shao

China

Wang Shutang

China

Jaswinder Singh

India

Mukesh Singh

United States of America

Helmut Sinzinger

Austria

George Siontis

Greece

Michael Skilton

Australia

Michelle Snyder

United States of America

Dragana Sobic-Saranovic

Serbia

Steinar Solberg

Norway

Stella Stabouli

Greece

Anthony Staines

Ireland

Andrew Steer

Australia

Debbie Stein

United States of America

Konstantinos Stellos

Germany

Rainer Storb

United States of America

Isabella Sudano

Switzerland

Hari Sundar

United States of America

Annelie Sundler Johansson

Sweden

Gulten Tacoy

Turkey

Marijana Tadic

Serbia

Johanna Takkenberg

Netherlands

Yodo Tamaki

Japan

Qizhu Tang

China

Ri-Bo Tang

China

Marco Tangelder

Netherlands

Ashis Tayal

United States of America

Elmarie Terblanche

South Africa

Gregor Theilmeier

Germany

Timothy Thiruchelvam

United Kingdom

Henry Thode

United States of America

Marjaana Tiainen

Finland

Emmi Tikkanen

Finland

Hermann Toplak

Austria

Yvan Torrente

Italy

Miyuki Tsuchihashi-Makaya

Japan

Karam Turk-Adawi

United States of America

Andrew Turley

United Kingdom

Ioanna Tzoulaki

Greece

Shiro Uemura

Japan

Joan Vaccaro

United States of America

Christopher Valerio

United Kingdom

Natalia Vallianou

Greece

Pieter van de Woestijne

Netherlands

Rob van der Pijl

Netherlands

Olivier M Van der Veken

Belgium

Alison Venn

Australia

Paulina Vermunt

Netherlands

Emilie Vierron

France

Veronica Ines Volberg

Argentina

Niels Wacher

Mexico

Yi Wan

China

Hongyu Wang

China

Kai Wang

China

Zhiguo Wang

Canada

Lisa Ware

South Africa

Ralf Wassmuth

Germany

Shipeng Wei

China

Raimund Weitgasser

Austria

Gillian Whalley

New Zealand

Bo Gregers Winkel

Denmark

Barbara Wizner

Poland

Wiktoria Wojciechowska

Poland

Dennis Wong

Australia

Nerys Woolacott

United Kingdom

Sean Wu

United States of America

Bo Xu

China

Hiromi Yamamoto

Japan

Shengkai Yan

China

Baofeng Yang

China

Guanyu Yang

China

Guohua Yang

China

Xingwei Zhe

China

jianming Zhi

China

Paul Ziegler

United States of America

Stefan Zimmermann

Germany

## Pre-publication history

The pre-publication history for this paper can be accessed here:

http://www.biomedcentral.com/1471-2261/14/17/prepub

